# *De novo* assembly of the Japanese lawngrass (*Zoysia japonica* Steud.) root transcriptome and identification of candidate unigenes related to early responses under salt stress

**DOI:** 10.3389/fpls.2015.00610

**Published:** 2015-08-20

**Authors:** Qi Xie, Jun Niu, Xilin Xu, Lixin Xu, Yinbing Zhang, Bo Fan, Xiaohong Liang, Lijuan Zhang, Shuxia Yin, Liebao Han

**Affiliations:** ^1^Institute of Turfgrass Science, College of Forestry, Beijing Forestry UniversityBeijing, China; ^2^Lab of Systematic Evolution and Biogeography of Woody Plants, College of Nature Conservation, Beijing Forestry UniversityBeijing, China; ^3^Bioinformatics, College of Plant Protection, Hunan Agricultural UniversityChangsha, China; ^4^Shenzhen Tourism College, Jinan UniversityShenzhen, China

**Keywords:** *Zoysia japonica* Steud., RNA sequencing (RNA-Seq), salt-stress, transcription factor, simple sequence repeats (SSRs)

## Abstract

Japanese lawngrass (*Zoysia japonica* Steud.) is an important warm-season turfgrass that is able to survive in a range of soils, from infertile sands to clays, and to grow well under saline conditions. However, little is known about the molecular mechanisms involved in its resistance to salt stress. Here, we used high-throughput RNA sequencing (RNA-seq) to investigate the changes in gene expression of *Zoysia* grass at high NaCl concentrations. We first constructed two sequencing libraries, including control and NaCl-treated samples, and sequenced them using the Illumina HiSeq™ 2000 platform. Approximately 157.20 million paired-end reads with a total length of 68.68 Mb were obtained. Subsequently, 32,849 unigenes with an N50 length of 1781 bp were assembled using Trinity. Furthermore, three public databases, the Kyoto Encyclopedia of Genes and Genomes (KEGG), Swiss-prot, and Clusters of Orthologous Groups (COGs), were used for gene function analysis and enrichment. The annotated genes included 57 Gene Ontology (GO) terms, 120 KEGG pathways, and 24 COGs. Compared with the control, 1455 genes were significantly different (false discovery rate ≤0.01, |log_2_Ratio |≥1) in the NaCl-treated samples. These genes were enriched in 10 KEGG pathways and 73 GO terms, and subjected to 25 COG categories. Using high-throughput next-generation sequencing, we built a database as a global transcript resource for *Z. japonica* Steud. roots. The results of this study will advance our understanding of the early salt response in Japanese lawngrass roots.

## Background

Plant growth, development, and production depend largely on soil; however, more than 800 million hectares of land worldwide are subjected to salt stress. High-salinity soils prevent the reabsorption of water from plant roots, the first organ that responds to salt stress (Zhu, 2002; Bartels and Sunkar, 2005; Munns, 2005; Petricka et al., 2012); therefore plants can be poisoned by the excess uptake of salts. The level of sensitivity of the plant salt stress response plays a crucial role in determining resistance to high salt stress (Tracy et al., 2008; Schmidt et al., 2013; Wei et al., 2013). Given the current scarcity of water resources, recycled water, and water use have been the focus of many studies (Marinho et al., 2013). Improving the abiotic stress resistance of plants is an effective method for overcoming water shortages and for the cultivation of large areas of saline land.

A focus on early detection would assist the determination of plant stress responses (Schmidt et al., 2013). Model plants such as rice have evolved a regulatory network of early responses to chilling stress (Yun et al., 2010). As a vital signaling component in many biological processes (Mittler et al., 2004; Schippers et al., 2012), reactive oxygen species (ROS) can rapidly induce abiotic stress responses—H_2_O_2_ levels induced by salt stress rise within several minutes in rice (Hong et al., 2009). Understanding the physiological and molecular mechanisms underlying salt stress tolerance in halophytes is therefore of great importance. Several halophytes, including *Thellungiella halophila, Mesembryanthemum crystallinum, Suaeda*, and *Populus*, have been studied (Dyachenko et al., 2006; Sun et al., 2010; Fukao et al., 2011; Yoon et al., 2014); however, our understanding of the unique tolerance mechanisms of halophytes is limited. Thus, further study of various halophytes may provide new information regarding the ability of plants to resist salt damage.

*Zoysia* Willd (family *Poaceae*, subfamily *Chloridoideae*, tribe *Zoysieae*) are perennial grasses, consisting globally of about 10 recognized species (Tsuruta et al., 2011). *Zoysia* grasses are widely used as a warm-season turfgrass for home lawns, golf courses, athletic fields, and parks (Ge et al., 2006). The species belonging to this genus are the most salt and cold tolerant of the C_4_ grass species in the family *Poaceae*. They are indigenous to China, Japan, and Korea. As one of the three most important commercial species, *Zoysia japonica* Steud. also exhibits marked tolerance to abiotic stress. It is distributed naturally in mountainous areas, along riversides, and in coastal areas, and it shows moderate tolerance to weak shade. Further, it can survive in various soils, ranging from infertile sands to clays (Tsuruta et al., 2011).

Few genomic sequence resources are available for *Zoysia* grasses. Only 11 mRNA sequences (complete cDNAs) are available in the database of the National Center for Biotechnology Information (NCBI) for *Z. japonica* (as of April 2015). This grass has an advanced transformation system (Asano, 1989; Inokuma et al., 1998; Ge et al., 2006), but the lack of sequence resources has limited the exploitation of *Z. japonica*'s genetic resources. To determine the molecular mechanism underlying the salinity tolerance of plants (Uddin et al., 2012), a large-scale analysis of differentially expressed genes (DEGs) is necessary.

Sequencing technology, which is used to analyze the transcriptome of a species without complete genome information, has undergone rapid progress in recent years with respect to the number of base calls and cost. Therefore, the technology is used in studies of many species and by researchers in many fields; for example, it has been used to study tissue regeneration in newts (Looso et al., 2013), glucosinolate metabolism in radish (Wang et al., 2013), and the leaf transcriptome of *Camelina sativa* (Liang et al., 2013).

In this study, we used the HiSeq™ 2000 platform to perform RNA sequencing (RNA-seq) of *Zoysia* grass roots. We compared the transcriptomes of plants grown under saline and normal conditions to identify differences in gene expression, and to identify the function of key transcripts and genes in the initiation of ROS-related signal transduction. Significant expression differences were found among genes involved in many metabolic pathways. Many novel genes were also identified and inferred to be expressed, specifically in the salt-treated plants. To the best of our knowledge, this is the first report of a transcriptome of *Zoysia* grass. We used H_2_O_2_ as a marker to identify DEGs between the control and salt-treated plants. This will improve our understanding of the mechanisms involved in short-term stress responses of *Zoysia*. These sequence data may also enhance our understanding of the molecular mechanisms in plants under salt stress, and provide a public dataset for use in future studies of *Zoysia*.

## Materials and methods

### Plant materials and treatment

*Z. japonica* Steud. cv. Zenith was grown from seed in soil in a propagation tray. Three weeks after germination, individual seedlings were transplanted to pots (diameter: 15 cm, depth: 14.5 cm) filled with a mixture of topsoil and coarse river sand (1:1) in a greenhouse (25°C during the day/20°C at night, 16 h of light/8 h of dark, 800 μmol m^−2^s^−1^ photosynthetically active radiation, and 75% relative humidity). The plants were irrigated with water during the first 3 weeks to keep the soil moist. After that, they were transferred to pots and maintained with 1/2 Murashige and Skoog cultivation solution once a week and water irrigation twice a week. NaCl treatment (150 mM) was initiated 3 months after germination.

### H_2_O_2_ assay

To visualize H_2_O_2_ accumulation, samples (from control and salt-treated plants) were immediately placed in a 1 mg/ml 3,3′-diaminobenzidine (DAB)-HCl solution in 10 mM phosphate buffer (pH 3.8) at 25°C for 4 h in the dark. After staining, the root of each plant was boiled in 95% (v/v) ethanol for 30 min and rehydrated in 70% (v/v) ethanol for 48 h at 25°C. H_2_O_2_ was visualized as a reddish-brown coloration. Each experiment was repeated using 10 plants.

### RNA preparation and library preparation for analysis

Total RNA was extracted using TRIzol reagent according to the manufacturer's instructions (Invitrogen, Carlsbad, CA, USA). The extracted RNA was treated with RNase-free DNase I (Takara Inc., Kyoto, Japan) for 45 min at 37°C to remove residual DNA. The quality of the RNA was evaluated using a NanoDrop 2000. The cDNA was prepared by pooling 10 μg of RNA each from the control and salt-treated samples.

Total RNA was extracted from normal and salt-treated plant roots. Poly A+ mRNA was obtained using a NEBNext Poly(A)mRNA Magnetic Isolation Module. Then, according to the instructions of the NEBNext mRNA Library Prep Master Mix Set for Illumina and NEBNext Multiplex Oligos for Illumina, a mixed cDNA library of salt-stressed (Case) seedlings and control (CK) plants was prepared.

### Sequencing, *de novo* assembly, and annotation

The cDNA library was sequenced using the Illumina HiSeq™ 2000 platform. After cleaning the raw reads and discarding low-quality reads, we ran Trinity (Yoon et al., 2014) to assemble the clean reads into transcripts. The clean reads were used to construct a K-mer dictionary and each K-mer was used as an initial contig. The most frequent K-mer, which was in the dictionary with K-1 overlaps with the current contig end, was extended until neither direction could be extended further. The contigs that shared at least one K-1-mer were then read across the junction sites to build a pool. Each contig was used to construct a de Brujin graph with trimmed spurious edges and compacted linear paths. It then reconciled the graph with reads and pairs, and output one linear sequence for each splice form and/or paralogous transcript represented in the graph. The final selection of the most important section from the partial transcript was the unigene. To annotate sequences obtained by *de novo* assembly, we used BLASTX with a significance threshold of *E*≤10^−5^. The assembly unigenes were aligned against the plant protein datasets of the non-redundant protein (NR), Swiss-prot protein, TrEMBL, Gene Ontology (GO), Clusters of Orthologous Groups (COGs), and Kyoto Encyclopedia of Genes and Genomes (KEGG) databases.

### Identification and functional annotation of DEGs

We calculated the expression levels as reads per kilobase exon model per million mapped reads (RPKM) for each root sample. DEGseq software was then used to determine significant DEGs defined as a fold change ≥2 and false discovery rate (FDR) < 0.01. Using BLAST, DEGs were aligned against the NR, GO, COGs, and KEGG databases.

### Gene comparison and sequence alignment within relative species

We previously analyzed published results on the salt response of model plants that are most homologous to *Zoysia*, including *Setaria*, barley, rice, and maize (Ueda et al., 2004, 2006; Qing et al., 2009; Puranik et al., 2011). We used Blastn with a significance threshold of *E* ≤ 10^−10^, sequence identified ≥30% and the length ≥30 aa. The DEGs were blasted against the salt-response genes that were identified in relative plants.

### Identification of *zoysia* transcription factors (TFs)

The TF database was downloaded from PlanTFDB (http://plntfdb.bio.uni-potsdam.de/v3.0/downloads.php). All unigenes were searched against those in the TF database by BLASTx (*E* < 10E^−5^).

### Quantitative reverse transcription PCR (RT-qPCR) verification

We subjected nine ERF salt stress-related unigenes (Comp132815_c0, Comp211710_c0, Comp196695_c0, Comp233109_c5, Comp223421_c0, Comp217707_c0, Comp206878_c0, Comp206878_c1, and Comp195676_c0) to RT-qPCR analysis. Root samples were collected from additional plants grown and tested under identical conditions. As per the Invitrogen protocol, total RNA was extracted from the root samples and then subjected to reverse transcription. The primers were designed using an online tool (http://www.idtdna.com/site). *ZjActin* (GenBank: GU290545.1) was used as a housekeeping gene. RT-qPCR was performed on a Bio-Rad RCR platform using a SYBR® Green Real-Time PCR Mix (TransStart Green qPCR SuperMix) to detect transcript abundance. Amplification was performed as follows: denaturation at 95°C for 30 s, followed by 40 cycles of denaturation at 95°C for 5 s, annealing at 60°C for 15 s, and extension at 72°C for 10 s. All amplifications were performed with three replicates. We calculated the relative expression levels of the selected unigenes using the 2^−Δ*ΔCt*^ method. The data from the reactions were analyzed using Bio-Rad software.

### Phylogenetic tree construction

Each of nine ERFs had more than one homologous genes in the PlnTFDB database. We selected 21 homologous genes (Supplementary File 1) from that database to perform phylogenetic analysis. Multiple sequence alignments were performed using the Clustal X program with default parameters. Phylogenetic analysis was performed by aligning a maximum-likelihood phylogenetic tree using MEGA 6 software, which was supported by a bootstrap test with 1000 iterations.

### Putative molecular markers

To investigate the distribution of simple sequence repeats (SSRs), the MIcroSAtellite identification tool (MISA version 1.0; http://pgrc.ipk-gatersleben.de/misa) was used to search the assembled transcripts. We scanned and counted the SSRs in all selected unigenes. The parameters were adjusted for the identification of perfect mono-, di-, tri-, tetra-, and penta-nucleotide motifs with a minimum of 12, 6, 5, 5, and 4 repeats, respectively.

## Results and discussion

### Physiological responses in the roots of salt-treated plants

Most previous studies focused on long-term adaptations to salt stress in grasses (Wang and Jiang, 2007; Bian and Jiang, 2009; Du et al., 2009; Hu et al., 2012); few have investigated the initiation of the stress response that plays an important role in this type of stress signaling. Changes in the environment of plants increase the level of ROS (Hong et al., 2009), with the production of ROS being an early response to abiotic stress (Zhu, 2002; Mittler et al., 2004). H_2_O_2_ is the predominant salt-induced ROS (Yang et al., 2007); thus, it was used as a marker of the degree of salt stress in root samples.

**Figure 1:**
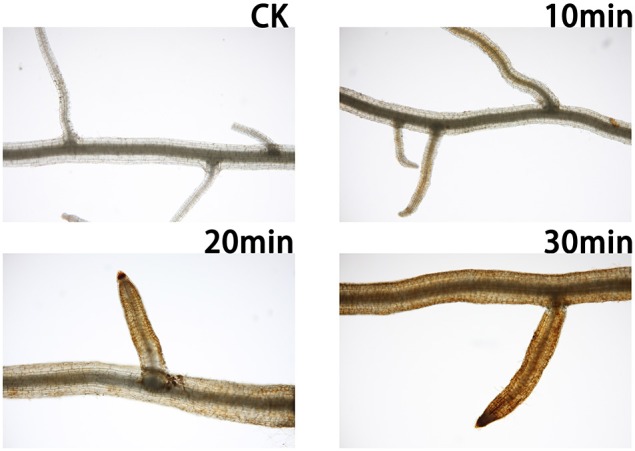
**Sample roots were treated with DAB to detect H_2_O_2_ in the dark for 4 h**. CK, Plants grown under normal conditions produced little H_2_O_2_; 10 min, in plants treated with salt for 10 min, the H_2_O_2_ level was slightly increased in lateral roots, while H_2_O_2_ was also detected in the main root; 20 min, in plants treated with salt for 20 min, H_2_O_2_ accumulation was detected in the entire root; 30 min, in plants treated with salt for 30 min, the roots were submerged in H_2_O_2_.

As shown in Figure 1, the H_2_O_2_ concentration in roots increased shortly after salt treatment. Compared with the control plants, a yellow substance was visible on the roots. After 30 min of salt treatment, oxidative stress caused by H_2_O_2_ was visible throughout the roots. Thus, we selected representative samples at 0 and 30 min for transcriptomic sequencing.

### Illumina sequencing and *de novo* assembly of *zoysia* root transcripts

RNA-seq technology is an indispensable tool for the whole-genome analysis of complex stress treatments. Compared with traditional large-scale sequencing, *de novo* whole-genome analysis is less costly and more efficient. It is suitable for those plants whose genomic sequences are unknown. We used RNA sequencing to analyze the transcriptome of *Z. japonica* Steud. roots.

To obtain a comprehensive transcriptome, two cDNA libraries denoted “CK” and “Case” prepared from three repeat RNA samples from normal and salt-treated roots were subjected to paired-end read sequencing using the Illumina platform. Paired-end read technology increases the depth and improves *de novo* assembly efficiency. Sequencing using the Illumina HiSeq™ 2000 platform resulted in the generation of 21.30 G raw reads. More than 80% had Phred-like quality scores at the Q30 level (error < 0.1%). After removing reads with adaptors, reads with unknown nucleotides, and low-quality reads, we obtained 105.47 million clean reads with an average GC content of 55.83% (Table 1A).

**Table 1:** **Summary of RNA-seq and ***de novo*** assembly of ***Zoysia japonica*** Steud**.

**A: STATISTICS OF OUTPUT SEQUENCING**							
**Sample ID**	**Clean reads**	**Raw reads**	**GC (%)**	**N (%)**	**Q20%**	**CycleQ20%**	**Q30%**
CK	24905482	5028905784	55.93	0.02	89.12	100.00	80.76
CASE	27691310	5593229543	55.87	0.02	89.20	100.00	80.89
**B: STATISTICS OF ASSEMBLY QUALITY**							
			**Contigs**				**Unigenes**
Total length (nt)			144,962,078				36,384,486
Total number (nt)			719,182				32,849
N50			243				1781
Mean length (nt)			201.56				1107
100–200 bp			592,688 (82.41%)				–
200–500 bp			83,393 (11.59%)				12,350 (37.59%)
500–1000 bp			23,577 (3.27%)				7276 (22.15%)
1000–1500 bp			8688 (1.20%)				4560 (13.88%)
1500–2000 bp			4933 (0.68%)				3439 (10.47%)
2000+			5903 (0.82%)				5224 (15.90%)

**Figure 2:**
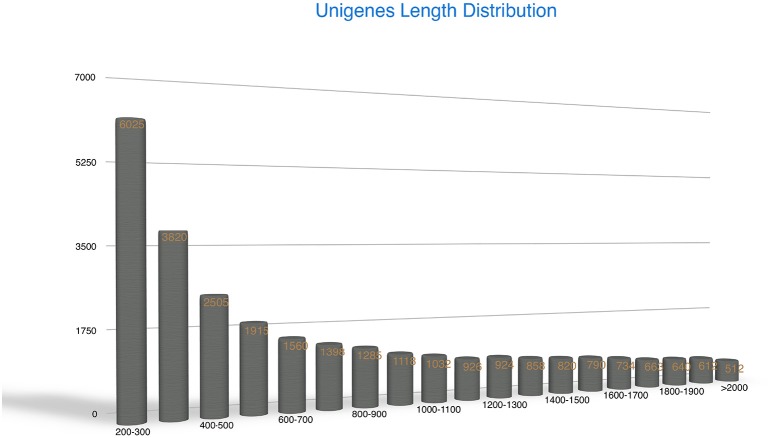
**Random distribution of the assembled unigenes**. The x-axis indicates the length of the unigenes. The y-axis indicates the number of unigenes.

The Trinity method can generate full-length transcripts without reference genomes (Grabherr et al., 2011; Haas et al., 2013). Using this method for *de novo* assembly, all high-quality clean reads were assembled into 719,182 contigs (Supplementary File 2) with an average length of 201.56 bp. Contigs of 100–500 bp were most frequent, accounting for 94% of the total. Subsequently, the contigs were clustered into 32,849 unigenes of which the mean length was 1107 bp and the N50 value was 1781 bp (Table 1B). There were 20,499 unigenes of ≥500 bp, and 5224 unigenes of ≥2000 bp. Most unigenes were in the range 200–500 bp (37.59%). Among these genes, the longest and shortest were 15,772 and 201 bp, respectively. The unigene lengths facilitated annotation and classification. The random distribution of the unigenes is presented in Figure 2.

### Functional annotation and classification of assembled unigenes

**Figure 3:**
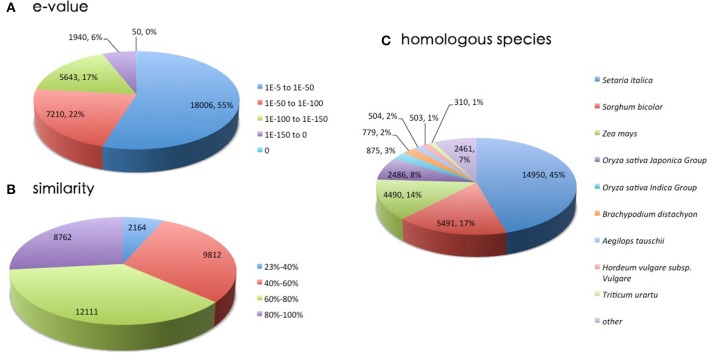
**A homology search was conducted by BLASTx against the NR database**. **(A)** *E*-value distribution of BLAST hits for matched unigene sequences. **(B)** Similarity distribution of top BLAST hits for each unigene. **(C)** Species distribution of the top BLAST hits.

The assembled sequences were first searched against the NR protein database, and analysis indicated that 55% of the sequences ranged from 1.0E^−5^ to 1.0E^−50^, while 18,006 of sequences with an *E* < 10E^−50^ displayed strong homology (Figure 3). The distribution of identity is shown in Figure 3B. The majority pattern was 60–80% similarity (12,111) and 40–60% similarity (9812). The closest species was *Setaria itallica*, with 14,950 genes (45%) matched. The next closest species was *Sorghum bicolor*, which showed 17% homology with *Zoysia*. This implies that the transcripts were assembled and annotated correctly (Dang et al., 2013; Wang et al., 2013). Figure 3C shows homologies with plants in the family Poaceae. Recent research (Ahn et al., 2015) supports our data showing that the most homologous model species to *Zoysia* is *Setaria itallica*, followed by *Sorghum bicolor* and *Zea mays*. Due to the differences in tissue between cultivar and treatment, our results were not completely consistent with that research.

GO annotation can provide a standardized vocabulary for assigning the functions of uncharacterized sequences. Based on sequence homology, 21,188 unigenes were categorized into 57 GO terms (Supplementary File 3) (Harris et al., 2004). The most frequently identified unigenes classified in these categories were “response to stimulus” (9918), “biological regulation” (8558) and “response to stress” (7371). A total of 13,480 unigenes were assigned to the COG classification (Supplementary File 3). There were 24 COG classes. The largest group was “general function prediction only” (2325, 17.24%), followed by “translation, ribosomal structure, and biogenesis” (1645, 12.20%), and “Posttranslational modification, protein turnover, chaperones” (1433, 10.63%). The smallest groups were “nuclear structures” (1, 0.0074%), and “cell motility” (25, 0.18%).

### Identification of DEGs

**Figure 4:**
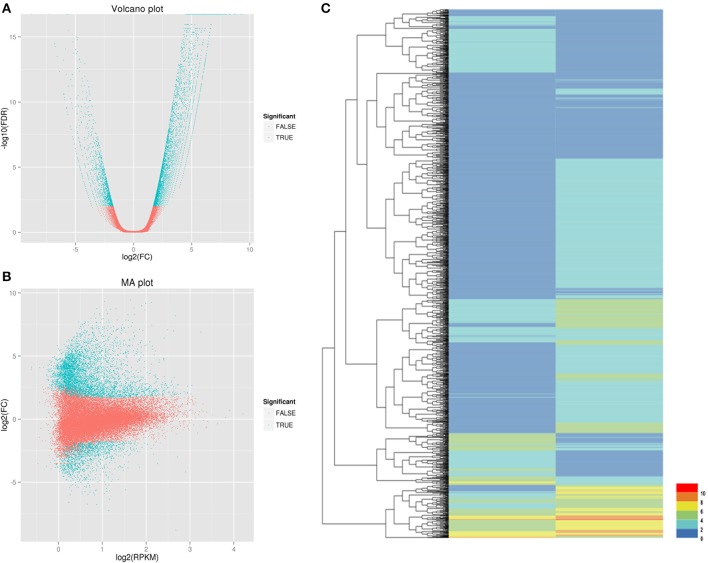
**Identification of DEGs between the CK (control) and Case (salt-stressed) samples**. **(A)** Volcano plot: The x-axis is the log of the fold change between the two conditions; the y-axis is the negative logarithm of the FDR. **(B)** MA plot: The x-axis is the average expression (log scale) level between the two conditions, indicating the basal expression level. The y-axis is the fold change (log scale), which indicates the difference between the two. **(C)** Each column represents a different sample. Each line represents a different gene. Each color represents a different gene expression level.

We used the RPKM method (RPKM > 0.1) to calculate the expression levels of unigenes in the control and salt-treated samples. Using DESeq software (FDR < 0.01, FC > 2) to analyze the DEGs between salt- and control-treated root samples, a total of 1648 differentially expressed unigenes (Figure 4) were obtained. Among those genes, 948 were upregulated and 700 were downregulated (Supplementary File 4).

### GO classification

**Figure 5:**
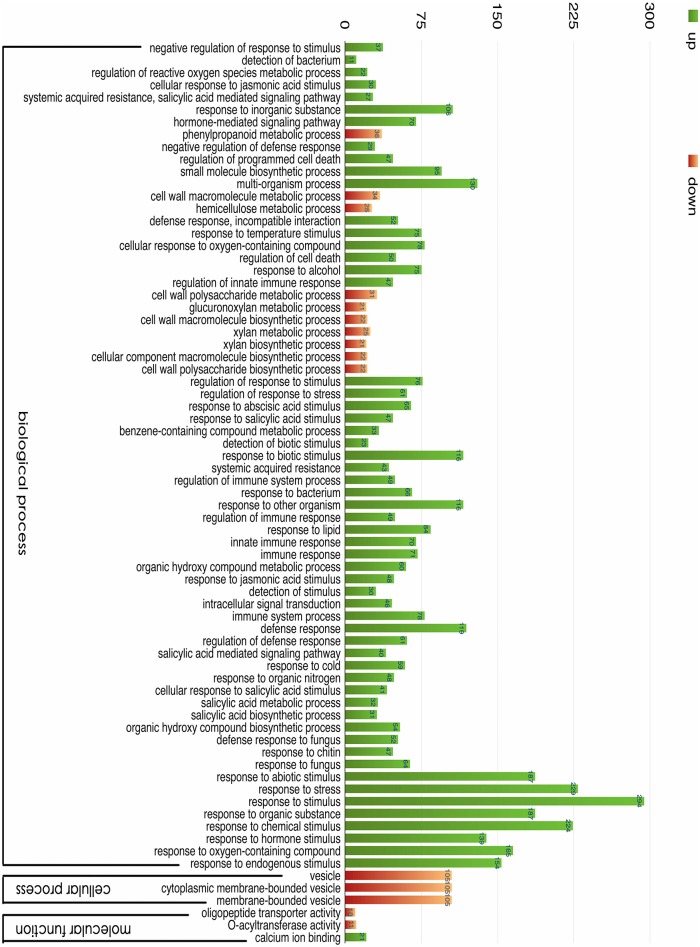
**GO classification of the unigenes**. The results are summarized for three main categories: biological process, cellular component, and molecular function. In total, 1445 DEGs with BLAST matches to known proteins were assigned to GO categories.

To categorize unigenes functionally, we assigned GO terms (*P* < 0.01). In total, 1,455 unigenes were enriched in 73 GO terms (Figure 5, Supplementary File 5). Among the unigenes, 798 were upregulated, and 647 were downregulated. After analyzing the statistically enriched GO functions related to DEGs, the majority of GO terms were assigned to biological processes distributed in 67 subcategories (91.78%), followed by cellular components and metabolic processes, with three subcategories each (4.11%).

The most over-represented GO terms in response to salt stress were “response to stimulus” (294 unigenes), “response to stress” (229 unigenes), “response to chemical stimulus” (224 unigenes), “response to abiotic stimulus” (187 unigenes), and “response to an organic substance” (187 unigenes). Many genes in these GO terms responded to short-term salt stress, suggesting that they play important roles in the early salt response. We also found nine significantly enriched hormone-related GO terms, including ABA-related (“response to abscisic acid stimulus”), JA-related (“response to jasmonic acid stimulus” and “cellular response to jasmonic acid stimulus”), and SA-related (“response to salicylic acid stimulus,” “cellular response to salicylic acid stimulus,” “salicylic acid-mediated signaling pathway,” “salicylic acid metabolic process,” “salicylic acid biosynthetic process,” “systemic acquired resistance,” and “salicylic acid-mediated signaling pathway”) terms. These hormones were upregulated by salinity, and they induced genes involved in alleviating salt stress (Wang et al., 2001). A GO terms analysis may help in understanding the salt tolerance mechanism involved when plants suffer a sudden increase in soil salinity.

### Calcium ion (Ca^2+^) signaling

Ca^2+^ acts as a secondary messenger in salt stress responses (Xuan et al., 2013). The cytosolic free Ca^2+^ concentration ([Ca^2+^]_*cyt*_) changes within seconds in plants subjected to salt stress (Knight et al., 1997), leading to downstream signaling. RSA1, a nuclear Ca^2+^-binding protein, can sense changes in the free Ca^2+^ level elicited by salt stress in the nucleus and transduce this signal by activating the *SOS1* promoter (Guan et al., 2013). P-type Ca^2+^ channels can sense changes in the [Ca^2+^]_*cyt*_ and transduce the signal downstream by activating specific targets (Huda et al., 2013). Among the DEGs, we identified 12 unigenes belonging to a major Ca^2+^ sensor family; of these, 10 were upregulated and two were downregulated (Table 2A).

**Table 2:** **List of some early salt stress response genes**.

**Unigenes ID**	**FDR**	**FC**	**Regulated**	**NR_annotation**
**(A) CALCIUM SIGNALING PATHWAY CALCIUM-SENSING PATHWAY**				
comp233131_c0	0.00415129	1.960818589	Up	CBL-interacting protein kinase 2-like
comp231679_c1	0.001561119	−1.993658032	Down	CBL-interacting protein kinase 4-like
comp236502_c0	9.83E-05	2.275044706	Up	CBL-interacting protein kinase family protein
comp231679_c0	1.39E-05	−2.762210406	Down	CBL-interacting protein kinase 4-like
comp178769_c0	5.68E-07	2.7825971	Up	Calcium-transporting ATPase 1, P-type
comp234684_c0	4.94E-06	2.613497363	Up	Calcium-dependent protein kinase 1-like
comp228160_c0	0.003964679	1.830598862	Up	Cation/calcium exchanger 1-like
comp225829_c0	1.78E-05	2.458344884	Up	Calcium-dependent protein kinase family protein
comp194938_c0	0.001820331	1.918099652	Up	Calcium-binding protein CML17-like
comp231687_c2	5.96E-06	2.50954593	Up	Calcium-binding protein CML50-like
comp213844_c0	0.00044765	2.078606714	Up	Calcium-binding protein CML22-like
comp212368_c0	1.19E-06	2.650783423	Up	Calcium-binding protein CML21-like
**(B) ROS-SCAVENGING**				
**Glutaredoxin (GLR)**				
comp173119_c0	0.000123406	−2.291632589	Down	Monothiol glutaredoxin-S2-like
comp210008_c0	0.000147098	−2.492488837	Down	Glutaredoxin-C7-like
**Thioredoxin (Trx)**				
comp232265_c1	0.009041204	−1.834919614	Down	Thioredoxin-like 3-3-like
comp225387_c1	6.84E-05	−2.644699895	Down	TPR repeat-containing thioredoxin TTL1-like
**Glutathione S-transferase (GST)**				
comp164920_c0	0.008757985	−1.962508223	Down	Glutathione S-transferase GSTU1-like
comp216567_c0	0.007836816	−3.16530996	Down	Glutathione S-transferase-like
**Peroxidase (POD)**				
comp209678_c1	0.004344984	1.813487744	Up	Peroxidase 52-like
comp209678_c0	5.06E-05	2.760100381	Up	Peroxidase 52-like
comp217804_c1	0.002697057	1.943654018	Up	Peroxidase 2
comp214494_c0	0.004313804	−3.727528392	Down	Peroxidase 2-like
comp207315_c0	0.005024896	−2.972862742	Down	Peroxidase 57-like
comp128226_c0	9.69E-05	−3.107721456	Down	Peroxidase 1-like
comp231079_c5	0.001558903	−2.054544569	Down	Peroxidase 5-like
comp215050_c0	1.60E-05	−3.32765436	Down	Peroxidase 2-like

### ROS scavenging

Salt stress can cause the rapid accumulation of ROS, including superoxide, H_2_O_2_, and hydroxyl radicals. ROS can perturb cellular redox homeostasis leading to DNA damage and changes in protein or membrane function. Plants possess ROS scavenging systems to control ROS levels and cope with oxidative stress. *Z. japonica* has a similar ROS scavenging system to that of other plant species. Cold treatment significantly increases antioxidant enzyme levels (Xuan et al., 2013), which may provide protection against oxidative damage in this species (Xu et al., 2013a). Among our DEGs, we identified 14 unigenes belonging to the ascorbate-glutathione cycle, GPX pathway, and Prx/Trx pathway. Among these unigenes, three were upregulated while 11 were downregulated (Table 2B).

### Comparison of salt-responsive genes in *zoysia* and relative species

To further characterize the identified genes, we used BLAST to compare 255 DEGs (Table 3, Supplementary File 6) with previously identified salt-responsive genes in four relative of the model plants. Genes in barley and rice had similar DEGs, and more than 60% of these DEGs were homologous sequences with *Zoysia*. Only 27 of 296 genes in maize were matched with those in *Zoysia*. These results suggest that barley and *Zoysia* use a similar mechanism to respond to salt stress, whereas maize has a different system for this response.

**Table 3:** **Comparison of salt response genes identified in this study with those in C4 model-plant**.

**Species**	**Tissue**	**Report genes**	**Overlap genes**	**Matching rate (%)**	**Treatment (mM)**	**Time course (h)**	**Report**
Balery-1	Leaf, root	92	62	67.39	200	1, 24	Ueda et al., 2006
Balery-2	Leaf, root	48	47	97.91	200	1, 24	Ueda et al., 2004
Maize	Leaf, root	296	27	9.12	100	5, 24, 48, 72	Qing et al., 2009
Rice	Leaf, root	42	33	78.57	150	1, 24	Ueda et al., 2006
Foxtail Millet	Whole plant	160	86	53.75	250	6	Puranik et al., 2011

### Major regulators of the salt-stress response transcriptome

Over the past decade, genes related to abiotic stress have been discovered and functionally characterized. Among these genes, TFs are master regulators that control gene clusters (Zhang et al., 2011). A single TF can regulate the expression of downstream genes by binding specifically to a *cis*-acting element in the promoter of a target gene. Members of the *AP2/ERF, MYB, WRKY*, and *NAC* families were shown to regulate salt tolerance. Enhanced expression of *DREB2A* can improve salt-stress tolerance in rice. Three rice *NAC* TFs (*SNAC1, SNAC2*, and *NAC5*) act as positive regulators of the salt stress response (Schmidt et al., 2013). A total of 3751 unigenes (Table 4) were identified as potential TFs with an average length of 1224 bp. The length distribution of the TFs is shown in Table 3. More than 43.05% of the TFs were longer than 1000 bp; the shortest TF was 201 bp, while the longest was 11,427 bp. In total, 80 TF families were identified (Supplementary File 7). The largest group was the *AP2/EREBP* family (187 unigenes), followed by the bHLH family (158 unigenes) and the WRKY family (137 unigenes). There were nine TF families containing more than 100 unigenes.

**Table 4:** **Unigene against TF database**.

***E*-values**	**TFs (%)**	**Identity (%)**	**TFs (%)**
1E-5 to 1E-50	2305 (61.45)	75–80	57 (1.51)
1E-50 to 1E-100	575 (15.33)	80–85	816 (21.75)
1E-100 to 1E-150	322 (8.58)	85–90	1397 (37.24)
1E-150 to 0	118 (3.14)	90–95	942 (25.11)
0	431 (11.49)	95–100	539 (14.37)
**Length distribution**			**TFs (%)**
200–300 bp			641 (17.08%)
300–400 bp			385 (10.26%)
400–500 bp			281 (7.49%)
500–600 bp			221 (5.89%)
600–700 bp			181 (4.82%)
700–800 bp			169 (4.50%)
800–900 bp			155 (4.13%)
900–1000 bp			103 (2.74%)
>1000 bp			1615 (43.05%)
Minimum length			201
Maximum length			11427
Average length			1224
Total TF numbers			3751

We selected the TF family that was most closely related to abiotic stress from the DEGs (Supplementary File 8).

### The *AP2/EREBP* family

Increasing evidence indicates that ERF proteins are involved in the salt response. They adjust the expression of downstream genes and fine-tune crosstalk between signaling pathways (Agarwal et al., 2006; Nakano et al., 2006; Fukao et al., 2011). Many studies have shown that ERFs enhance salt tolerance (Gilmour et al., 2000; Park et al., 2001; Jung et al., 2007). Among the unigenes we identified were 187 ERF genes, including 34 DEGs. About 33 DEGs were upregulated and only 1 DEG (comp223372_c0) was downregulated. In both libraries, comp232359_c0 had the highest expression level, but the expression of this gene was slightly increased in the case libraries. The largest change was found for comp234321_c1, which was upregulated more than four-fold (log_2_ fold change).

#### The *bZip* family

Members of the *bZIP* family of TFs respond to abiotic stress. They have important roles in the ABA response and the regulation of oxidative and pathogen defense responses (Yun et al., 2010). In total, 115 genes were identified in our database, with seven DEGs. Comp219239_c0 had a higher expression level, and its expression differed slightly between the two sample libraries.

#### The *NAC* family

The NAC domain, which was identified based on consensus sequences from the Petunia NAM, *Arabidopsis* ATAF1/2, and CUC2 proteins, was plant-specific and contained the highly conserved NAC DNA-binding domain and variable C-terminal domains. The *NAC* family is considered to be important in plant development, senescence, auxin responses, and biotic stress responses (Aida et al., 1997; Kim et al., 2004; Olsen et al., 2005; Lu et al., 2007; Hao et al., 2011; Nakashima et al., 2012; Sun et al., 2012). After salt treatment, the expression of 131 *NACs* differed, and only 11 unigenes were considered to be DEGs. The transcript of comp231035_c0 was present at a high level in both libraries, and was upregulated almost two-fold under salt stress.

#### The *WRKY* family

As one of the best-characterized TF families, the *WRKY* TF family has been suggested to play an important role in plant stress responses. The *WRKY* protein family contains a conserved amino acid sequence motif, *WRKYGOK*, at the N-terminus and a novel zinc-finger-like motif at the C-terminus. Numerous studies have demonstrated that *WRKY* family regulates various plant processes from development to biotic and abiotic stress responses (e.g., wounding, drought, and salinity). Eight *WRKYs* in wheat respond to low temperature, salt stress, and heat treatment (Wu et al., 2008). Enhancing the expression of *WRKY25* and *WRKY33* can increase the salt tolerance of *Arabidopsis thaliana* (Jiang and Deyholos, 2009; Li et al., 2011). The overexpression *GmWRKY54* increases salt and drought tolerance in soybean. H_2_O_2_ treatment significantly induces the expression of *AtWRKY30, AtWRKY75, AtWRKY48, AtWRKY39, AtWRKY6, AtWRKY53*, and *AtWRKY22* (Vanderauwera et al., 2005; Miao and Zentgraf, 2007; Zhou et al., 2011). In our study, we identified 137 *WRKYs*, including 20 DEGs. Of these, seven *WRKYs* (comp231105_c0, comp224918_c0, comp232021_c2, comp230256_c0, comp216034_c0, comp218505_c0, and comp190635_c0) had high transcription levels in both libraries.

#### The *MYB* family

MYB proteins are the largest class of TFs in plants. Nearly 9% of the TFs in *Arabidopsis* are *MYBs*, and more than 1600 TFs have been identified (Riechmann et al., 2000). MYB proteins have DNA-binding domains, which contain one to four imperfect repeats in plants. Many studies have shown that *MYBs* are involved in abiotic stress responses. *AtMYB41* is induced by drought, during which it modulates cell expansion and cuticle deposition (Cominelli et al., 2008). The overexpression of *MdoMYB121* in tomato and apple confers improved tolerance to drought (Cao et al., 2013). *TaMYB73* has been shown to enhance salt resistance by regulating the expression of related genes (He et al., 2012). A total of 129 genes were identified; among them, only 14 showed significant differences in expression.

### The *bHLH* family

TFs belonging to the bHLH family are important in plant development, circadian regulation, and stress responses. Here, we found 158 *bHLH* genes; 19 genes from this family were DEGs.

### Putative molecular markers

Molecular markers are important for molecular breeding (Liu et al., 2012; Chen et al., 2013, 2014; Dai et al., 2013; Xu et al., 2013b; Li et al., 2014). SSRs are 1-6-bp iterations of DNA sequences that occur only in noncoding regions. The occurrence of SSRs in transcribed sequences has been established. The roles of SSRs in plants such as rice (Cho Yg et al., 2000), bread wheat (Gupta et al., 2003), and sugarcane (Cordeiro et al., 2001) have been reported.

In our study, 32,849 unigenes were used to detect SSRs, and a total of 4842 SSRs were identified in 3951 unigenes using MISA (Supplementary File 9). Among them, 702 unigenes contained at least 2 SSRs. The largest fraction was mono-nucleotide SSRs (2305), followed by tri-nucleotide SSRs (1694), and di-nucleotide SSRs (778). This phenomenon corresponds to natural selection. Molecular markers are a useful resource for determining functional genetic variation. Our results will facilitate the prediction of molecular markers for *Zoysia*.

## Analysis of genes of interest

### RT-qPCR

**Figure 6:**
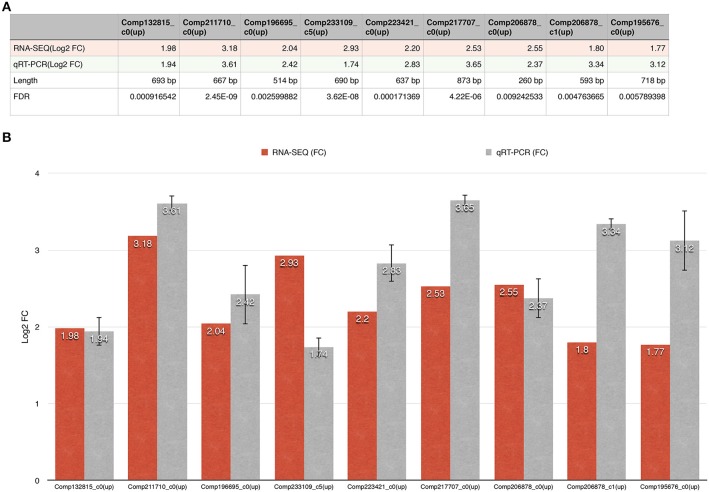
**The fold changes in the DEGs as determined using RNA-seq and RT-qPCR are shown. (A)** The first line represents the unigene ID; the second line represents the fold change in the DEG; the third line represents the RT-qPCR result; the fourth line represents the length of the unigene; and the fifth line represents the FDR of the unigene. **(B)** The x-axis represents the unigene ID while the y-axis represents the fold change in expression of the unigenes.

We identified nine ERFs with significant changes; their lengths varied from 260 to 873 bp (Figure 6). PCR amplification showed that all RT-qPCR primers (Supplementary File 10) used produced only single fragments of the expected lengths (100–250 bp; 100% success rate). All positive clones from the validation studies were subjected to Sanger sequencing, and the results confirmed those obtained using the Illumina method. Our qPCR results for the nine unigenes are in agreement with those from the DESeq analysis of our RNA-seq data.

### Phylogenetic analysis

To further predict and distinguish the function of the nine unigenes, phylogenetic analysis was performed using 21 known plant AP2/EREBP protein sequences that were homologous to these unigenes. The analysis revealed the presence of five distinct clusters (Supplementary File 11), suggesting that these five groups might differ functionally in some respect. Comp206878_c1, Orza_sativa_subsp._indica_OsIBC009072, and Zea_mays_GRMZM2G129674_P01 belong to class I, whereas comp196695_c0, Zea_mays_GRMZM2G175525_P01, and Sorghum_bicolor_5041828 belong to the class II. In the phylogenetic tree, comp132815_c0, comp223421_c0, and comp211710_c0 were not closely related to the known AP2/EREBP genes, and comp233109_c5, comp217707_c0, comp195676_c0, and comp206878_c0 had a low bootstrap value. If these seven unigenes are novel ERFs or novel genes, the full lengths of these unigenes can be used further to inform our phylogenetic tree.

## Conclusions

Here, we presented the first comprehensive transcriptome data of *Z. japonica* Steud. roots. In total, 32,849 unigenes were identified. The large number of transcripts identified will serve as a global resource for future studies. TFs play a crucial role in the early response to salt stress; thus, we identified candidate TFs related to salt stress, which will be the subject of future studies. In addition, a total of 4842 SSRs were identified. This information will facilitate future studies of plant biology and molecular breeding.

## Author contributions

HL conceived and designed the experiments and contributed the reagents. XQ and NJ wrote the manuscript. XQ, ZY, and FB performed the experiments. XX conducted the TF analysis. YS conducted the DEG analysis. ZL and SX conducted the H_2_O_2_ assay. XL and LX provided the materials and analytic tools.

### Conflict of interest statement

The authors declare that the research was conducted in the absence of any commercial or financial relationships that could be construed as a potential conflict of interest.

## Supplementary material

Supplementary File 1 **Detail of the 21 known AP2/EREBP genes**.

Supplementary File 2 **Length frequency distribution of contigs obtained by *de novo* assembly**.

Supplementary File 3 **COG and GO classification of zoysiagrass transcriptome**.

Supplementary File 4 **Summary and functional annotation of the identified DEGs**.

Supplementary File 5 **Gene ontology classification of DEGs**.

Supplementary File 6 **Detail of salt related genes that from homologous species**.

Supplementary File 7 **Transcript distribution across TF families**.

Supplementary File 8 **Major TFs identified in the DEG database**.

Supplementary File 9 **SSRs derived from the identified unigenes**.

Supplementary File 10 **Primers used for experimental validation**.

Supplementary File 11 **Phylogenetic tree of nine ERFs**.

## Availability of supporting data

The RNA sequence dataset supporting the results in this article is available from the NCBI Sequence Read Archive. The accession number is SRX838917.
