# Corrigendum: *De novo* assembly of the Japanese lawngrass (*Zoysia japonica* Steud.) root transcriptome and identification of candidate unigenes related to early responses under salt stress

**DOI:** 10.3389/fpls.2015.00811

**Published:** 2015-10-09

**Authors:** Qi Xie, Jun Niu, Xilin Xu, Lixin Xu, Yinbing Zhang, Bo Fan, Xiaohong Liang, Lijuan Zhang, Shuxia Yin, Liebao Han

**Affiliations:** ^1^Institute of Turfgrass Science, College of Forestry, Beijing Forestry UniversityBeijing, China; ^2^Lab of Systematic Evolution and Biogeography of Woody Plants, College of Nature Conservation, Beijing Forestry UniversityBeijing, China; ^3^Bioinformatics, College of Plant Protection, Hunan Agricultural UniversityChangsha, China; ^4^Shenzhen Tourism College, Jinan UniversityShenzhen, China

**Keywords:** *Zoysia japonica* Steud., RNA sequencing (RNA-Seq), salt-stress, transcription factor, simple sequence repeats (SSRs)

Due to an oversight by the authors, data from a different study were incorrectly reported in the Abstract and Conclusion of the manuscript.

The number of unigenes found was 32,849, not the 100,800 originally reported. This has been changed throughout the article. Accordingly, the abstract should read as follows:

We first constructed two sequencing libraries, including control and NaCl-treated samples, and sequenced them using the Illumina HiSeq™ 2000 platform. Approximately 157.20 million paired-end reads with a total length of 68.68 Mb were obtained. Subsequently, 32,849 unigenes with an N50 length of 1781 bp were assembled using Trinity, among which 70,127 unigenes were functionally annotated (*E*-value ≤ 10-5) in the non-redundant protein (NR) database. Furthermore, three public databases, the Kyoto Encyclopedia of Genes and Genomes (KEGG), Swiss-prot, and Clusters of Orthologous Groups (COGs), were used for gene function analysis and enrichment. The annotated genes included 57 Gene Ontology (GO) terms, 120 KEGG pathways, and 24 COGs. Compared with the control, 1455 genes were significantly different (false discovery rate ≤0.01, |log2Ratio|≥1) in the NaCl-treated samples. These genes were enriched in 10 KEGG pathways and 73 GO terms, and subjected to 25 COG categories.

The conclusion has been modified to remove the text “of which 73,862 produced hits against the NR database” as this was in reference to the incorrect data.

All conclusions were drawn using the correct data and, as such, these are unaffected by the above changes.

The original article was updated.

## Conflict of interest statement

The authors declare that the research was conducted in the absence of any commercial or financial relationships that could be construed as a potential conflict of interest.

