# Supplementary figures and images for: De novo assembly of the Japanese lawngrass (Zoysia japonica Steud.) root transcriptome and identification of candidate unigenes related to early responses under salt stress
Source: Front Plant Sci. 2015 Aug 20;6:610. doi: 10.3389/fpls.2015.00610 (PMC4542685; doi:10.3389/fpls.2015.00610)

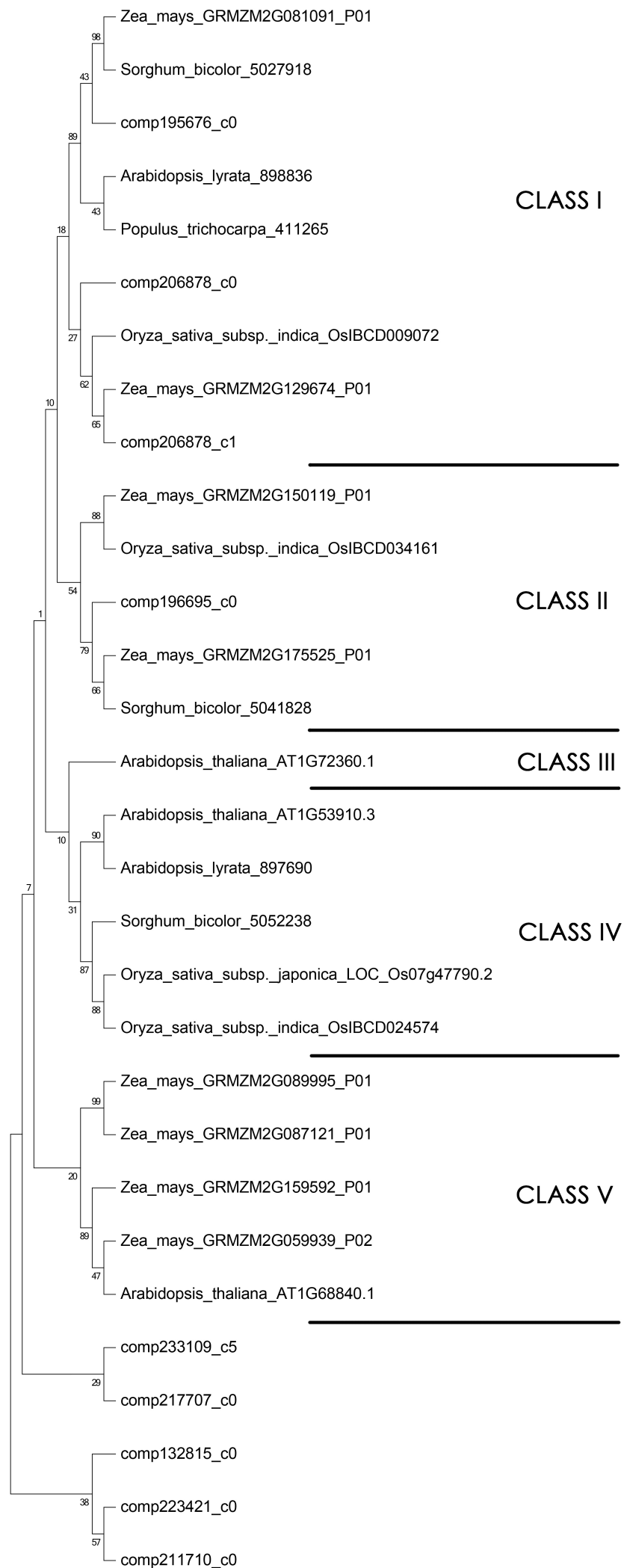

Supplement: Supplementary File 11 — Phylogenetic tree of nine ERFs. [file DataSheet11.PDF]
